# ONTO-ToolKit: enabling bio-ontology engineering via Galaxy

**DOI:** 10.1186/1471-2105-11-S12-S8

**Published:** 2010-12-21

**Authors:** Erick Antezana, Aravind Venkatesan, Chris Mungall, Vladimir Mironov, Martin Kuiper

**Affiliations:** 1 Department of Biology, Norwegian University of Science and Technology (NTNU), Høgskoleringen 5, N-7491 Trondheim, Norway; 2 Lawrence Berkeley National Laboratory, 1 Cyclotron Road, Berkeley, CA 94720, USA

## Abstract

**Background:**

The biosciences increasingly face the challenge of integrating a wide variety of available data, information and knowledge in order to gain an understanding of biological systems. Data integration is supported by a diverse series of tools, but the lack of a consistent terminology to label these data still presents significant hurdles. As a consequence, much of the available biological data remains disconnected or worse: becomes misconnected. The need to address this terminology problem has spawned the building of a large number of bio-ontologies. OBOF, RDF and OWL are among the most used ontology formats to capture terms and relationships in the Life Sciences, opening the potential to use the Semantic Web to support data integration and further exploitation of integrated resources via automated retrieval and reasoning procedures.

**Methods:**

We extended the Perl suite ONTO-PERL and functionally integrated it into the Galaxy platform. The resulting ONTO-ToolKit supports the analysis and handling of OBO-formatted ontologies via the Galaxy interface, and we demonstrated its functionality in different use cases that illustrate the flexibility to obtain sets of ontology terms that match specific search criteria.

**Results:**

ONTO-ToolKit is available as a tool suite for Galaxy. Galaxy not only provides a user friendly interface allowing the interested biologist to manipulate OBO ontologies, it also opens up the possibility to perform further biological (and ontological) analyses by using other tools available within the Galaxy environment. Moreover, it provides tools to translate OBO-formatted ontologies into Semantic Web formats such as RDF and OWL.

**Conclusions:**

ONTO-ToolKit reaches out to researchers in the biosciences, by providing a user-friendly way to analyse and manipulate ontologies. This type of functionality will become increasingly important given the wealth of information that is becoming available based on ontologies.

## Background

Bio-ontologies are artefacts used to represent, build, store, and share knowledge about a biological domain by capturing the domain entities and their interrelationships. Bio-ontologies have become an important asset for the life sciences. They not only provide a controlled, standard terminology (to support annotations for instance); a variety of tools are available to exploit these ontologies, making them one of the cornerstones for biological data analysis. The Gene Ontology (GO) [1] is probably the best known bio-ontology. One of the most common uses of the GO is to perform term enrichment [2,3] on a gene set. The GO website lists over fifty such tools [4]. In addition, the life sciences community began to utilise other available ontologies (such as the Plant Ontology [5]) as well as to develop their own bio-ontologies to support other biology or technology domains. A recent example is the Ontology of Biomedical Investigations (OBI [6]), a community effort to build an ontology describing the different elements of a biomedical investigation (e.g. protocols, instruments, reagents, experimentalists). The Open Biomedical Ontologies (OBO) foundry [7] suggests a set of principles to guide the development of ontologies, for instance the ‘orthogonality principle’ designed to prevent overlapping ontologies. Most of the bio-ontologies gathered by the OBO foundry are represented in the OBO format [8], which has became the *lingua franca* to build bio-ontologies. An increasing number of bio-ontologies is being developed in the more expressive Web Ontology Language (OWL) that allows for advanced automated reasoning [9,10]. Automated reasoning, performed on OWL-formatted ontologies via the so-called reasoners (such as HermiT [11]), allows bio-ontologists to perform various tasks such as classification (also known as subsumption), which enables the process of making explicit the relations that were hidden (i.e. implicitly captured), and in general provides help to ensure the consistency of an ontology.

Several open source tools are available to deliver native support for bio-ontology manipulation (BioPerl [12], ONTO-PERL [13], BioRuby [14], BioPython [15]). We have previously published ONTO-PERL, a suite of Perl tools supporting the management of ontologies represented in OBO format (OBOF). ONTO-PERL is a full-blown API to manipulate bio-ontologies in OBOF. It offers a set of scripts supporting the typical ontology manipulation tasks, which can be used from the command line. Useful as this API may be for bioinformaticians or expert ontologists, biologists may find it intimidating to use. To accommodate their easy use, working with ontologies has for instance been facilitated by the setting up of ontology portals [16,17]. These applications can be directly linked to knowledge systems that store information in local infrastructures, thus taking advantage of the ontological scaffold (generally, hierarchical and partonomical relationships) through mappings between the ontology components (terms and relationships) and actual data. The linking of ontologies and biological data is proving to be a successful stepping stone towards ontology-based knowledge discovery platforms [18]. Those platforms may eventually become important tools in the quest for new hypotheses that can drive experimental design.

To further improve the repertoire of tools available to biologists to handle and analyze the knowledge available through ontologies we have turned to Galaxy [19], a web-based environment that integrates various types of tools to handle biological data. Galaxy’s development is strongly targeted towards end-users who have limited computational skills (including many molecular biologists), so that they may easily perform analysis or have their favourite command line tool integrated. A tracking of the history of analyses, support for building workflows and data sharing are among Galaxy’s most appealing features.

We used Galaxy to construct ONTO-ToolKit, which is an extension of the ONTO-PERL software that we developed previously. ONTO-PERL consists of a collection of Perl modules that enable the handling of OBO-formatted ontologies (like the Gene Ontology). With these modules a user can for instance manipulate ontology elements such as a Term, a Relationship and so forth, or employ scripts to carry out various typical tasks (such as format conversions between OBO and OWL (*obo2owl*, *owl2obo*).

ONTO-ToolKit allows exploiting the ONTO-PERL functionality within the Galaxy environment. Galaxy not only provides a user friendly interface to manipulate OBOF ontologies, it also offers the possibility to perform further biological (and ontological) analyses by using other tools provided within the Galaxy platform. In addition, ONTO-ToolKit provides tools to translate OBOF ontologies into Semantic Web formats such as RDF (Resource Description Framework) and OWL.

## Methods

**Figure 1:**
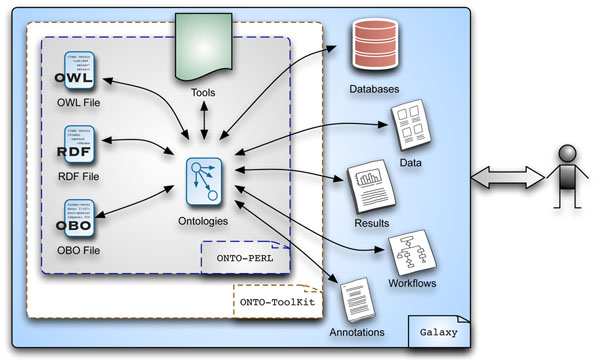
**Schematic representation of ONTO-PERL, ONTO-ToolKit and Galaxy.** The ONTO-ToolKit suite of tools provides a support within the Galaxy framework to analyse and manipulate OBO-formatted ontologies. ONTO-ToolKit relies on the functionality enabled by ONTO-PERL to handle bio-ontologies and to enable operations (such as format conversions from OBO to OWL) that could in turn produce results that might be further analysed and exploited through other tools (such as workflows or statistical analyses) provided in the Galaxy environment.

The functionalities of ONTO-PERL are enabled as tools in Galaxy through a set of tool configuration files (XML files), or ‘wrappers’. These files contain execution details of the tool, e.g. path to the script, the arguments and the output format. Table 1 lists the functionalities provided by ONTO-PERL that are useful to understand the relationship between various biological components. The script *get_ancestor_terms.pl*, for instance, retrieves all the ancestor terms for a particular term id from a given OBO ontology. Furthermore, through *obo2owl.pl* and *obo2rdf.pl* scripts users can convert their data (OBOF) into OWL and RDF, respectively. A schematic representation of how ONTO-PERL is embedded as ONTO-ToolKit in Galaxy is given in Figure 1. A detailed description of installing ONTO-ToolKit is available at http://bitbucket.org/easr/onto-toolkit/wiki/Home.

**Table 1:** Examples of ONTO-PERL functionalities

Scripts	Functionality
get_ancestor_terms.pl	Collects the ancestor terms (list of IDs) from a given term (existing ID) in the given OBO ontology.
get_child_terms.pl	Collects the child terms (list of term IDs and their names) from a given term (existing ID) in the given OBO ontology.
get_descendent_terms.pl	Collects the descendent terms (list of IDs) from a given term (existing ID) in the given OBO ontology.
get_subontology_from.pl	Extracts a sub-ontology (in OBO format) of a given ontology having the given term ID as the root.
get_obsolete_terms.pl	Finds all the obsolete terms in a given ontology.
get_parent_terms.pl	Collects the parent terms (list of term IDs and their names) from a given term (existing ID) in the given OBO ontology.
get_relationship_types.pl	Finds all the relationship types in a given ontology.
get_root_terms.pl	Finds all the root terms in a given ontology.
get_term_synonyms.pl	Finds all the synonyms of a given term name in an ontology.
get_terms.pl	Finds all the terms in a given ontology.
get_terms_by_name.pl	Finds all the terms in a given ontology that have a given string in their names.
obo2owl.pl	OBO to OWL translator.
obo2rdf.pl	OBO to RDF translator.
obo_trimming.pl	This script trims a given branch of OBO ontology.
obo2cco.pl	Converts an ontology into another one which could be integrated into CCO.
obo2tran.pl	OBOF into RDF translator. The resulting file has (full) transitive closure
obo2xml.pl	OBO to XML translator (CCO scheme).
go2owl.pl	Gene Ontology (in OBO) to OWL translator.
goa2rdf.pl	Generates a simple RDF graph from a given GOA file
owl2obo.pl	OWL to OBO translator.
obsolete_term_id_vs_def_in_go.pl	Obsolete terms vs. their definitions
obsolete_term_id_vs_name_in_go.pl	Obsolete terms vs. their names
term_id_vs_term_def.pl	Gets the term IDs and term definitions of a given ontology.
term_id_vs_term_name.pl	Gets the term IDs and term names of a given ontology.
term_id_vs_term_namespace.pl	Gets the term IDs and its namespaces in a given ontology
get_list_intersection_from.pl*	Collects common OBO terms from a given set of lists containing OBO terms
get_intersection_ontology_from.pl*	Provides an intersection of the given ontologies (in OBO format)

The left column lists ONTO-PERL scripts available in ONTO-ToolKit, with their functionality described in the right column. *: Scripts written specifically for ONTO-ToolKit and included in the ONTO-ToolKit download package.

## Results

We illustrate the use of ONTO-ToolKit through three ontology-analysis use cases. In use case I we have analysed the relationship between terms from the Cell Cycle Ontology (CCO), an application ontology that we described previously [20]. In use case II we carried out an analysis combining ONTO-ToolKit functionality with other tools available in Galaxy, and in use case III we have demonstrated how a workflow was built to analyse gene sets with GO and *S. pombe* annotations.

### Use case I: “Investigating similarities between given molecular functions”

**Figure 2:**
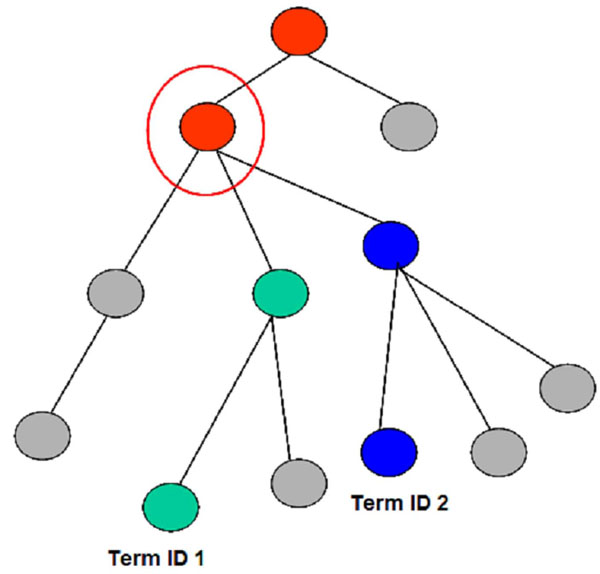
**Schematic diagram of use case I** The nodes and the edges represent a section of an ontology, with the higher nodes representing terms with general descriptions, and the nodes further down in the graph depicting terms with higher specificity. The nodes in green and blue represent the terms associated with the molecular function term id 1 and term id 2, respectively. The red nodes represent the terms shared by search terms, with the most specific term encircled in red.

**Figure 3:**
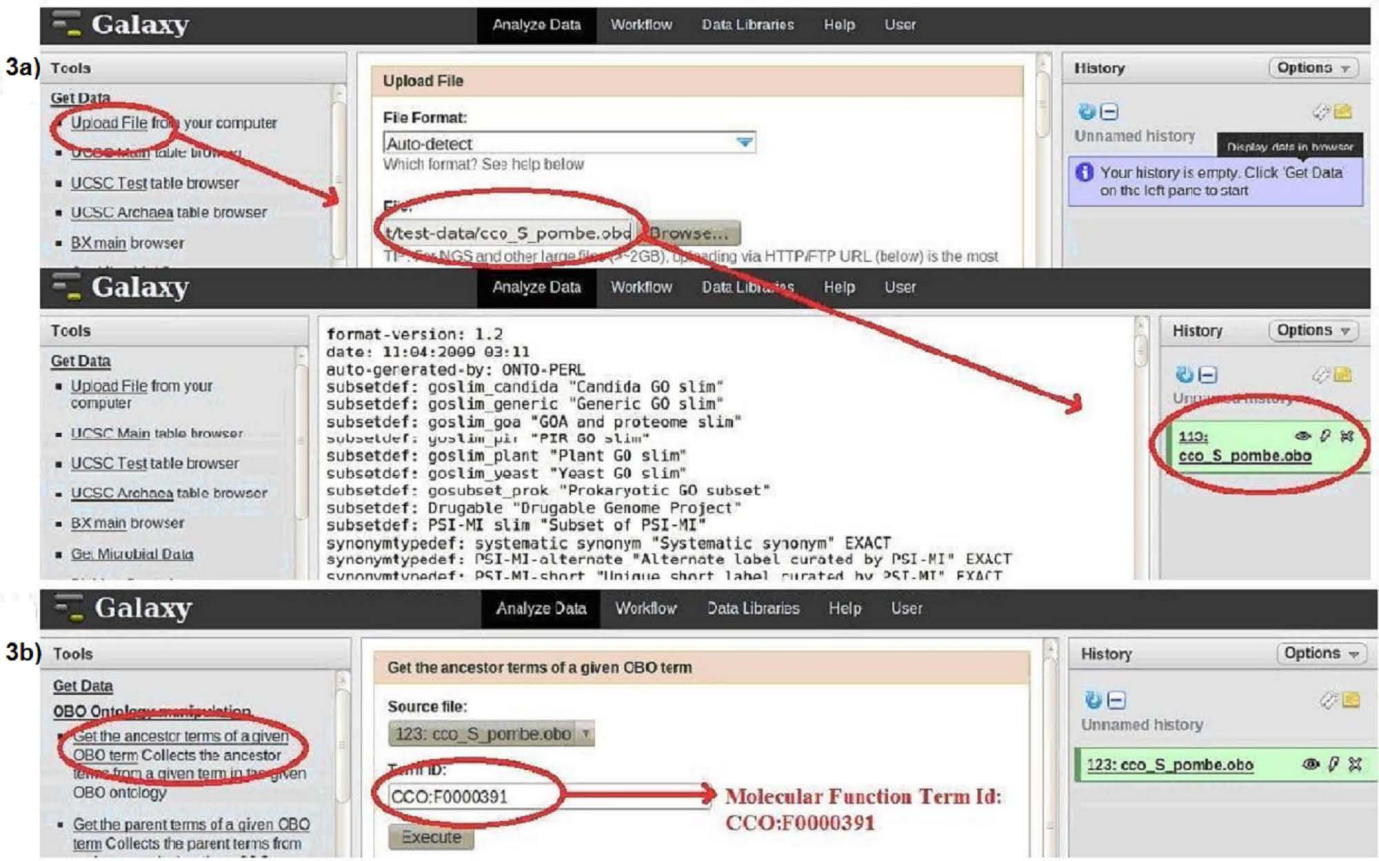
**Screenshot of use case I** implementation – step 1. Details of use case I analysis in the Galaxy user interface. **3a:** Method to upload the chosen obo ontology (CCO *S. pombe*). The uploaded ontology can be browsed, a feature available in Galaxy (encircled on the right); **3b:** Demonstration of the method to query the uploaded ontology using the *get_ancestor_term* function with the chosen term ID as the argument.

**Figure 4:**
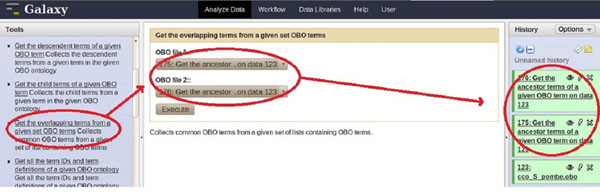
**Screenshot of step 2 in use case I** Illustration of use case I, step 2. The Galaxy interface shows the use of the *get_overlapping_terms* function to intersect the two sets of terms obtained in step 1.

The first use case illustrates the functionality of ONTO-ToolKit in identifying the ontology terms linking a pair of molecular function terms. A user might be interested to search for the most specific ancestor term that is shared by two molecular functions, to see if these functions fall into the same biological category. As a primary step all ancestor terms pertaining to the molecular function term IDs defined in a query are retrieved. In a next step a comparison is made between the two sets of ancestor terms for their relatedness. Figure 2 shows a schematic depiction of this use case, with retrieval of individual ancestor terms and checking for the most specific terms shared by the two molecular functions specified in the query. It is noteworthy that such a step will always result in a set of shared upper-level terms (as all molecular function terms are linked to the root), but obviously the relationship will be more specific if their shared terms are positioned further away from the root of the ontology, where information is more fine-grained. To implement this concept, the *S. pombe-*specific CCO was chosen along with the two molecular function term IDs (*CCO:F0000391* -- 6-phosphofructokinase activity; *CCO:F0000759*--glucokinase activity). The analysis consisted of several steps. Firstly, using the *get_ancestor_terms* functionality two queries were used to fetch the ancestor terms for each of the two term IDs (see Figure 3). This resulted in two sets of ancestor terms and annotations associated with the terms. The intersection of these two sets was determined using the *get_list_intersection_from* function yielding one set of specific terms (see Figure 4) and corresponding annotations allowing the assessment of the relatedness of the initial terms.

**Figure 5:**
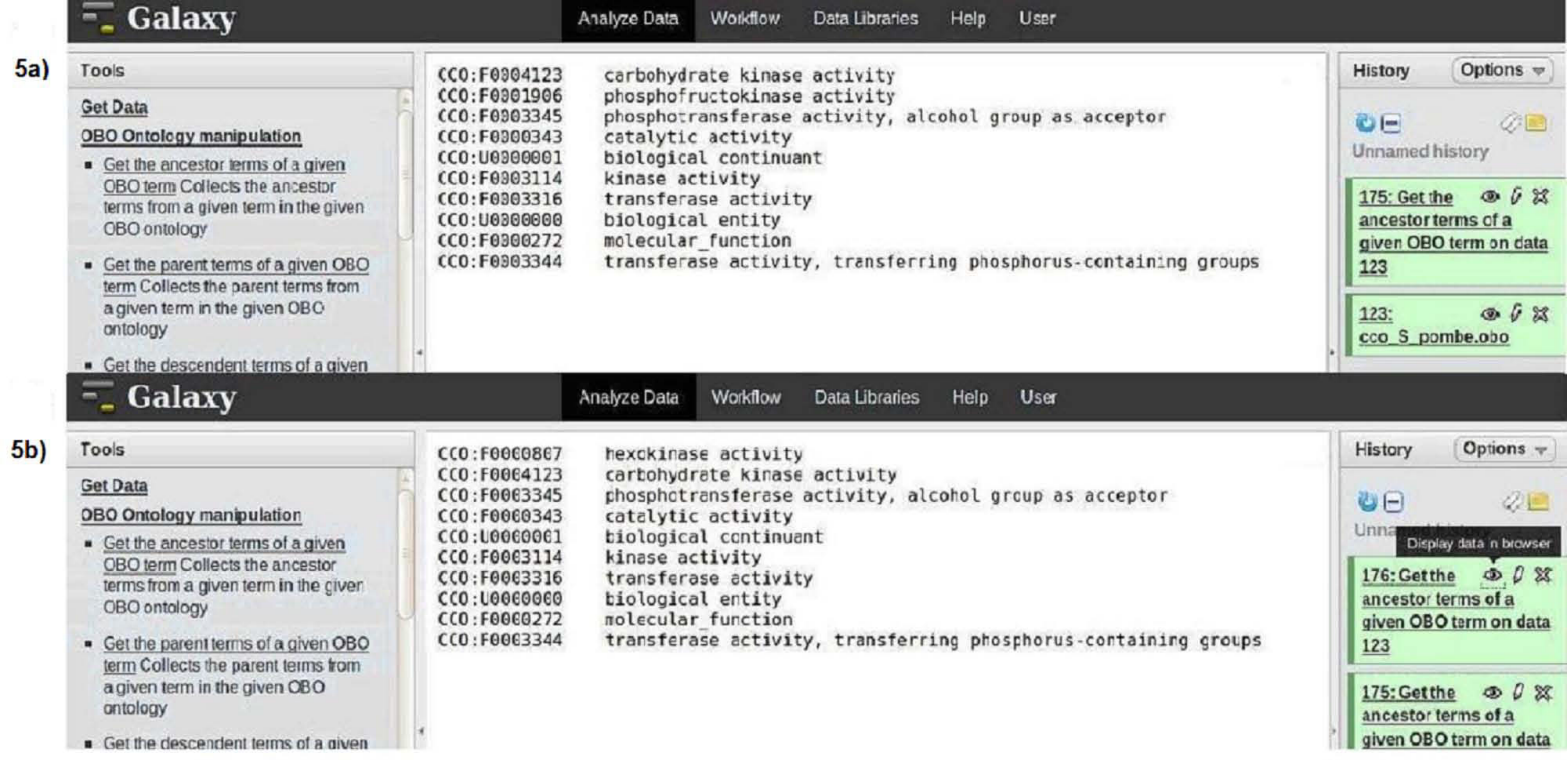
**Ancestors term list – use case I** Illustration of use case I, results. Panel 5a shows the results obtained for the term ID *CCO: F0000391*. Panel 5b shows ancestor terms for the term ID *CCO:F0000759*

**Figure 6:**
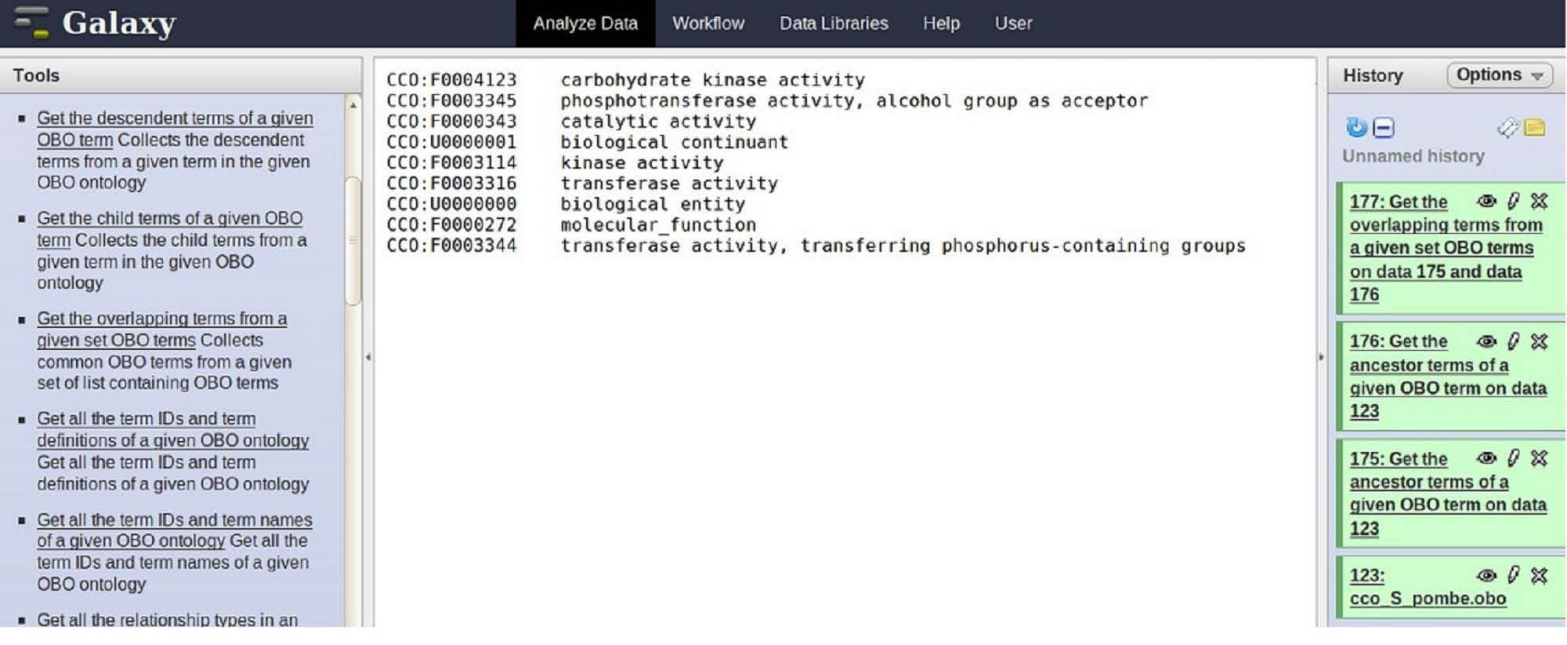
**Intersection of ancestor terms – use case I** Use case I results. The main panel shows the intersection of the two sets of ancestor terms of the terms of the query.

Figure 5 shows the set of ancestor terms for the two terms of the query. For both the terms (*CCO:F0000391, CCO:*F0000759**)** ten ancestor terms were retrieved (see Supplementary file). Furthermore, the most specific common terms for the two molecular function term IDs were retrieved (see Figure 6). This list (Additional file 1) contained nine terms that were common, with various degrees of specificity, to both the molecular function terms. The most specific terms shared between them were: *CCO:F0004123 – carbohydrate kinase activity, CCO:F0003345 – phosphotransferase activity, CCO:F0003344 – transferase activity*. These results suggest that the two chosen terms are related, and additional ancestral terms make it clear that the two molecular function terms both describe functions of the glycolytic pathway in *S. pombe*.

### Use case II: “Identifying shared terms for a pair of proteins”

Use case II illustrates how ONTO-ToolKit can be used in combination with other functionalities available in Galaxy. A user might be interested in identifying the functional relatedness of two proteins, as described by their GO annotations. To assess this, two lists of GO Terms associated with the two proteins need to be retrieved and then matched to determine their intersection. The example uses the *H. sapiens proteins JUN* (UniProt ID: P05412) and *FOS* (UniProt ID: P01100). Their UniProt IDs were used to query the BioMart [21] central server from within Galaxy to retrieve lists of *JUN* and *FOS* GO terms and annotations (see Additional file 1). In the second step, the ONTO-ToolKit function *get_list_intersection_from* was used to obtain all the annotations shared between *JUN* and *FOS* (see Additional file 1). The results show the four GO terms (GO:0010843, GO:0070412, GO:0060395, GO:0007179) common between these two transcription factors.

### Use case III: “Performing term enrichment using an ontology subset”

**Figure 7:**
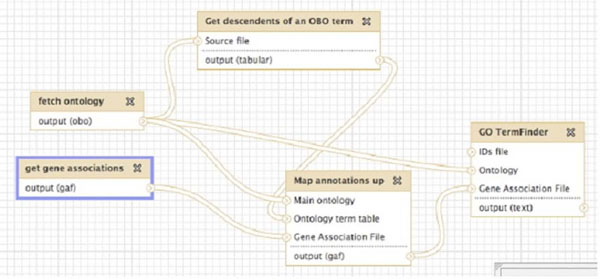
**Example of workflow in Galaxy** The boxes depict functions and intermediate workflow steps; the arrows indicate how these functions are connected.

Use case III shows how ONTO-ToolKit can be used to create interdependent workflows (see Figure 7). Here a researcher may wish to analyze an *S. pombe* gene expression dataset using a subset of GO. The dataset contains a set of genes that have a high likelihood of being differentially expressed, and the researcher wants to know if this gene set has an overrepresentation of GO terms that are annotated to a specific biological process. As this type of analysis considers all GO terms sequentially, running this analysis on the whole GO may result in insignificant P-values due to the large hypothesis space. This may be remedied by reducing this hypothesis space – for example, by considering only the role of these genes in the cell cycle.

This workflow starts by fetching an ontology and a set of gene associations, in this case, the Gene Ontology and the *S. pombe* annotations. The next step is to use the *get_descendant_terms* function (the converse of the *get_ancestor_terms* function described above) to extract a subset of the ontology (in this case, it is configured to extract all descendants of the term “cell cycle”). To get the corresponding annotations an annotation mapping function is used to get all annotations corresponding to this sub ontology. This cell cycle specific annotation file is fed into the GO TermFinder [3] enrichment tool, along with a user-supplied gene set. This workflow can be reused multiple times (for example, to re-check results with the latest ontology and annotations), and can be shared between Galaxy users.

## Discussion

A coherent integration of public, online information resources is still a major bottleneck in the post-genomic era. Bioinformatics databases are especially difficult to integrate because they are often complex, highly heterogeneous, dispersed and incessantly evolving [22-24]. Moreover, consensus naming conventions and uniform data standards are often lacking. Nevertheless, the need for efficient procedures to integrate data is only increasing, due to the growing popularity of integrative biology and systems biology: approaches that need a variety of data from multiple sources to build computational models in order to understand biological systems behaviour.

Bio-ontologies can greatly facilitate this integration process [25] because they provide a scaffold that allows computers to automate parts or the whole of the integration process [26]. Setting up an integrative platform that can support an advanced data analysis based on bio-ontologies typically requires the establishment of an environment that enables access both to the many public biological databases that contain curated information, and to the various bio-ontologies. Moreover, such an integrative environment must enable the sharing of the information at any time with all contributors to the data curation process. In addition to curated databases, vast amounts of literature-independent data are being generated by high-throughput genome-wide analyses and accumulated in various databases. These databases represent another resource of context to infer biological function and to assess relations between biological entities. To obtain a powerful structuring and synthesis of all available biological knowledge it is essential to build an efficient information retrieval and management system. This system requires an extensive combination of data extraction methods, data format conversions, ontology-based analysis support and a variety of information sources. Ultimately, such an integrated and structured knowledge base may facilitate the use of computational reasoning for analysis of biological systems, an approach that we have named Semantic Systems Biology [26].

ONTO-ToolKit offers functionality that allows a biologist to exploit the increasingly abundant information supported by ontologies. The Gene Ontology Consortium is participating in the development of ONTO-ToolKit as an integration platform for performing many GO based workflows, replacing existing functionality in AmiGO [27] and expanding the range of tools to be used. For example, it is possible to extract all experimental annotations for the clade Mammalia, generate a slim (subset) from this set, or to fetch all annotations belonging to a pre-defined ontology subset. Annotations extracted in this way can also be used in term enrichment analyses using GO TermFinder [3]. Term enrichment analysis on ontology subsets reduces the number of terms that are considered for the overrepresentation analysis, making the analysis more sensitive.

Platforms such as Galaxy are aimed to overcome the barriers in global data processing, and its flexibility offers ample opportunity to identify and implement new ways to fill the gaps in data visualisation and analysis. We have explored Galaxy’s use to implement data analysis techniques based on bio-ontologies. Bioinformatics data resources are constantly updated, *i.e.* by automated, software-mediated annotation or manual curation processes that depend on human intervention. Ontologies provide a means of improving the annotation process and to semantically represent the knowledge contained in biological databases in an unambiguous way. ONTO-ToolKit builds on this trend by enabling the manipulation of bio-ontologies within an integrative platform, which in turn allows analysis results to become the entry-point for further biological data analysis.

## Conclusions

We presented several use cases to illustrate how the functionality of ONTO-PERL can be combined with the functionality of other tools in Galaxy. We have shown how the functionality of ONTO-PERL can be used to identify all the ancestor terms of a pair of ontology terms, or to simply retrieve all the terms shared by two proteins in order to assess their potential biological relatedness. We have extended and used ONTO-ToolKit to build a workflow to dynamically extract a subset of GO, map annotations to this subset, and then perform term enrichment analysis. With this we have shown that ONTO-ToolKit constitutes a useful extension to the functionalities available in Galaxy, by adding a variety of ontology-based analysis approaches that can improve the depth of the overall analysis because it builds on an increasing wealth of annotation and curation results.

## Availability

ONTO-ToolKit can be obtained from its project page [28] or from the Galaxy Tool Shed [29]. ONTO-ToolKit is distributed under an Open Source License: GNU General Public License [30]. ONTO-ToolKit provides access to the latest obo2owl conversion code that implements the new proposed OBO Foundry mapping to OWL [31]. Once the ontology is converted to OWL, there are a number of OWL processing tools available, including Pellet [32], and ontology processing via the Thea library [33].  OntoToolkit, including the workflow example mentioned in use case III, is also available on-line [34].

## Competing interests

The authors declare that they have no competing interests.

## List of abbreviations used

OBO: Open Biomedical Ontologies; OBOF: Open Biomedical Ontologies Format; OBI: Ontology of Biomedical Investigations; GO: Gene Ontology; CCO: Cell Cycle Ontology; RDF: Resource Description Framework; OWL: Web Ontology Language; XML: eXtensible Markup Language.

## Authors' contributions

EA implemented the ONTO-PERL extensions, the ONTO-ToolKit tools and steered the project. AV implemented use cases I and II. CM implemented the workflow example. VM and MK provided expertise in biological data management. All the authors have contributed to and approved the manuscript.

## Supplementary Material

Additional file 1 This file contains all the additional results referred to in the description of the use cases I and II.
**Subsection I:** Use case I - Lists the ancestor terms for CCO:F0000391.
**Subsection II:** Use case I - Lists the ancestor terms for CCO:F0000759.
**Subsection III:** Use case I - Lists the overlapping terms generates as part of step 2.
**Subsection IV:** Use Case II - GO terms associated with JUN (Uniprot ID: P05412)
**Subsection V:** Use Case II - GO terms associated with FOS (Uniprot ID: P01100)
**Subsection VI:** Use Case II - Intersection of GO terms associated JUN and FOS Click here for file
