# Supplementary material for: ONTO-ToolKit: enabling bio-ontology engineering via Galaxy
Source: BMC Bioinformatics. 2010 Dec 21;11(Suppl 12):S8. doi: 10.1186/1471-2105-11-S12-S8 (PMC3040534; doi:10.1186/1471-2105-11-S12-S8)
Supplement: Additional file 1 — This file contains all the additional results referred to in the description of the use cases I and II. Subsection I: Use case I - Lists the ancestor terms for CCO:F0000391. Subsection II: Use case I - Lists the ancestor terms for CCO:F0000759. Subsection III: Use case I - Lists the overlapping terms generates as part of step 2. Subsection IV: Use Case II - GO terms associated with JUN (Uniprot ID: P05412) Subsection V: Use Case II - GO terms associated with FOS (Uniprot ID: P01100) Subsection VI: Use Case II - Intersection of GO terms associated JUN and FOS [file 1471-2105-11-S12-S8-S1.pdf]

Subsection I: Ancestor terms for CCO:F0000391 - 6-phosphofructokinase activity:

| | |
|---|---|
| CCO:F0004123 | carbohydrate kinase activity |
| CCO:F0001906 | phosphofructokinase activity |
| CCO:F0003345 | phosphotransferase activity, alcohol group as acceptor |
| CCO:F0000343 | catalytic activity |
| CCO:U0000001 | biological continuant |
| CCO:F0003114 | kinase activity |
| CCO:F0003316 | transferase activity |
| CCO:U0000000 | biological entity |
| CCO:F0000272 | molecular_function |
| CCO:F0003344 | transferase activity, transferring phosphorus-containing groups |

Subsection II: Ancestor terms for CCO:F0000759 - glucokinase activity:

| | |
|---|---|
| CCO:F0000807 | hexokinase activity |
| CCO:F0004123 | carbohydrate kinase activity |
| CCO:F0003345 | phosphotransferase activity, alcohol group as acceptor |
| CCO:F0000343 | catalytic activity |
| CCO:U0000001 | biological continuant |
| CCO:F0003114 | kinase activity |
| CCO:F0003316 | transferase activity |
| CCO:U0000000 | biological entity |
| CCO:F0000272 | molecular_function |
| CCO:F0003344 | transferase activity, transferring phosphorus-containing groups |

Subsection III: List of overlapping terms between CCO:F0000391 and CCO:F0000759

| | |
|---|---|
| CCO:F0004123 | carbohydrate kinase activity |
| CCO:F0003345 | phosphotransferase activity, alcohol group as acceptor |
| CCO:F0000343 | catalytic activity |
| CCO:U0000001 | biological continuant |
| CCO:F0003114 | kinase activity |
| CCO:F0003316 | transferase activity |
| CCO:U0000000 | biological entity |
| CCO:F0000272 | molecular_function |
| CCO:F0003344 | transferase activity, transferring phosphorus-containing groups |

Subsection IV: Use Case II - GO terms associated with JUN (Uniprot ID: P05412)

| GO ID | GO name |
|---|---|
| GO:0010843 | F:promoter binding |
| GO:0070412 | F:R-SMAD binding |
| GO:0005100 | F:Rho GTPase activator activity |
| GO:0003705 | F:RNA polymerase II transcription factor acti... |
| GO:0003713 | F:transcription coactivator activity |
| GO:0016564 | F:transcription repressor activity |
| GO:0043392 | P:negative regulation of DNA binding |
| GO:0043923 | P:positive regulation by host of viral transc... |
| GO:0045944 | P:positive regulation of transcription from R... |
| GO:0007184 | P:SMAD protein nuclear translocation |
| GO:0060395 | P:SMAD protein signal transduction |
| GO:0007179 | P:transforming growth factor beta receptor si... |
| GO:0010843 | F:promoter binding |
| GO:0070412 | F:R-SMAD binding |
| GO:0005100 | F:Rho GTPase activator activity |
| GO:0003705 | F:RNA polymerase II transcription factor acti... |
| GO:0003713 | F:transcription coactivator activity |
| GO:0016564 | F:transcription repressor activity |
| GO:0043392 | P:negative regulation of DNA binding |
| GO:0043923 | P:positive regulation by host of viral transc... |
| GO:0045944 | P:positive regulation of transcription from R... |
| GO:0007184 | P:SMAD protein nuclear translocation |
| GO:0060395 | P:SMAD protein signal transduction |
| GO:0007179 | P:transforming growth factor beta receptor si... |
| GO:0010843 | F:promoter binding |
| GO:0070412 | F:R-SMAD binding |
| GO:0005100 | F:Rho GTPase activator activity |
| GO:0003705 | F:RNA polymerase II transcription factor acti... |
| GO:0003713 | F:transcription coactivator activity |
| GO:0016564 | F:transcription repressor activity |
| GO:0043392 | P:negative regulation of DNA binding |
| GO:0043923 | P:positive regulation by host of viral transc... |
| GO:0045944 | P:positive regulation of transcription from R... |
| GO:0007184 | P:SMAD protein nuclear translocation |
| GO:0060395 | P:SMAD protein signal transduction |
| GO:0007179 | P:transforming growth factor beta receptor si... |
| GO:0010843 | F:promoter binding |
| GO:0070412 | F:R-SMAD binding |
| GO:0005100 | F:Rho GTPase activator activity |
| GO:0003705 | F:RNA polymerase II transcription factor acti... |
| GO:0003713 | F:transcription coactivator activity |
| GO:0016564 | F:transcription repressor activity |
| GO:0043392 | P:negative regulation of DNA binding |
| GO:0043923 | P:positive regulation by host of viral transc... |
| GO:0045944 | P:positive regulation of transcription from R... |
| GO:0007184 | P:SMAD protein nuclear translocation |
| GO:0060395 | P:SMAD protein signal transduction |
| GO:0007179 | P:transforming growth factor beta receptor si... |
| GO:0010843 | F:promoter binding |
| GO:0070412 | F:R-SMAD binding |

| | |
|---|---|
| GO:0005100 | F:Rho GTPase activator activity |
| GO:0003705 | F:RNA polymerase II transcription factor acti... |
| GO:0003713 | F:transcription coactivator activity |
| GO:0016564 | F:transcription repressor activity |
| GO:0043392 | P:negative regulation of DNA binding |
| GO:0043923 | P:positive regulation by host of viral transc... |
| GO:0045944 | P:positive regulation of transcription from R... |
| GO:0007184 | P:SMAD protein nuclear translocation |
| GO:0060395 | P:SMAD protein signal transduction |
| GO:0007179 | P:transforming growth factor beta receptor si... |
| GO:0010843 | F:promoter binding |
| GO:0070412 | F:R-SMAD binding |
| GO:0005100 | F:Rho GTPase activator activity |
| GO:0003705 | F:RNA polymerase II transcription factor acti... |
| GO:0003713 | F:transcription coactivator activity |
| GO:0016564 | F:transcription repressor activity |
| GO:0043392 | P:negative regulation of DNA binding |
| GO:0043923 | P:positive regulation by host of viral transc... |
| GO:0045944 | P:positive regulation of transcription from R... |
| GO:0007184 | P:SMAD protein nuclear translocation |
| GO:0060395 | P:SMAD protein signal transduction |
| GO:0007179 | P:transforming growth factor beta receptor si... |

Subsection V: Use Case II - GO terms associated with FOS (Uniprot ID: P01100)

| GO ID | GO name |
|---|---|
| GO:0010843 | F:promoter binding |
| GO:0070412 | F:R-SMAD binding |
| GO:0003704 | F:specific RNA polymerase II transcription fa... |
| GO:0003700 | F:transcription factor activity |
| GO:0034614 | P:cellular response to reactive oxygen species |
| GO:0006306 | P:DNA methylation |
| GO:0006954 | P:inflammatory response |
| GO:0060395 | P:SMAD protein signal transduction |
| GO:0007179 | P:transforming growth factor beta receptor si... |
| GO:0010843 | F:promoter binding |
| GO:0070412 | F:R-SMAD binding |
| GO:0003704 | F:specific RNA polymerase II transcription fa... |
| GO:0003700 | F:transcription factor activity |
| GO:0034614 | P:cellular response to reactive oxygen species |
| GO:0006306 | P:DNA methylation |
| GO:0006954 | P:inflammatory response |
| GO:0060395 | P:SMAD protein signal transduction |
| GO:0007179 | P:transforming growth factor beta receptor si... |
| GO:0010843 | F:promoter binding |
| GO:0070412 | F:R-SMAD binding |
| GO:0003704 | F:specific RNA polymerase II transcription fa... |
| GO:0003700 | F:transcription factor activity |
| GO:0034614 | P:cellular response to reactive oxygen species |
| GO:0006306 | P:DNA methylation |
| GO:0006954 | P:inflammatory response |
| GO:0060395 | P:SMAD protein signal transduction |
| GO:0007179 | P:transforming growth factor beta receptor si... |

Subsection VI: Use Case II - Intersection of GO terms associated JUN and FOS

| GO ID | GO name |
|---|---|
| GO:0010843 | F:promoter binding |
| GO:0070412 | F:R-SMAD binding |
| GO:0060395 | P:SMAD protein signal transduction |
| GO:0007179 | P:transforming growth factor beta receptor si... |
